# A Finite Element Model to Investigate the Stability of Osteochondral Grafts Within a Human Tibiofemoral Joint

**DOI:** 10.1007/s10439-024-03464-6

**Published:** 2024-03-06

**Authors:** Gavin A. Day, Alison C. Jones, Marlène Mengoni, Ruth K. Wilcox

**Affiliations:** Institute of Medical and Biological Engineering, Mechanical Engineering, University of Leeds, Leeds, UK

**Keywords:** Cartilage, Bone, Finite element analysis, Knee, Modelling, Osteoarthritis, Surgical repair

## Abstract

Osteochondral grafting has demonstrated positive outcomes for treating articular cartilage defects by replacing the damaged region with a cylindrical graft consisting of bone with a layer of cartilage. However, factors that cause graft subsidence are not well understood. The aim of this study was to develop finite element (FE) models of osteochondral grafts within a tibiofemoral joint, suitable for an investigation of parameters affecting graft stability. Cadaveric femurs were used to experimentally calibrate the bone properties and graft-bone frictional forces for use in corresponding image-based FE models, generated from µCT scan data. Effects of cartilage defects and osteochondral graft repair were measured by examining contact pressure changes using further in vitro tests. Here, six defects were created in the femoral condyles, which were subsequently treated with osteochondral autografts or metal pins. Matching image-based FE models were created, and the contact patches were compared. The bone material properties and graft-bone frictional forces were successfully calibrated from the initial tests with good resulting levels of agreement (CCC = 0.87). The tibiofemoral joint experiment provided a range of cases that were accurately described in the resultant pressure maps and were well represented in the FE models. Cartilage defects and repair quality were experimentally measurable with good agreement in the FE model pressure maps. Model confidence was built through extensive validation and sensitivity testing. It was found that specimen-specific properties were required to accurately represent graft behaviour. The final models produced are suitable for a range of parametric testing to investigate immediate graft stability.

## Introduction

Articular cartilage lesions can occur from trauma or degeneration and are associated with pain, joint instability, and progression to end-stage osteoarthritis (OA) [1]. Cartilage defects and lesions pose a significant challenge for clinicians [2], with current conservative treatments being costly and failing to address disease progression [3]. Surgical options for the treatment of cartilage defects aim to restore the articulating surfaces of the joint and include repair through microfracture, regeneration through autologous chondrocyte implantation, or replacement through osteochondral grafting. Only the latter procedure provides an immediate hyaline cartilage replacement with a continuous cartilage surface [4, 5].

The osteochondral grafting procedure and associated surgical instrumentation have a number of variants; however, the general aims of the treatment are to replace the area of damaged cartilage with an osteochondral graft containing a layer of healthy cartilage that is flush with the surrounding surface. While the technique has been used successfully clinically, literature describing the mechanical stability and properties is limited. Success depends on the grafts remaining flush with the surrounding cartilage while osseointegration stabilises the bone components of the graft [6]. The angle of implantation [7], depth of cartilage and bone [8–12], and method of implantation [13] have all been shown to affect the stability of the grafts or the resultant contact pressure on the cartilage surfaces. However, a general consensus for the correct grafting parameters has not been reached. This is especially true for property matching between the grafts and surrounding host bone, which is of particular importance not only for allografts but for the increasing number of synthetic and tissue engineered grafts that are currently in development.

Experimentally, graft stability and tribological performance can be measured through the use of uniaxial push-in tests [13, 14], reciprocating pin-on-plate friction tests [15] and tibiofemoral joint testing [16, 17]. However, there are a wide range of properties which can have large effects on the resultant behaviour of graft implantation, stemming from the variation in properties of bone and cartilage quality of the target, osteoarthritic demographic. Computational approaches may be used for investigating this variation using a parameterised approach [18, 19]; however, current studies have not used specimen-specific methodologies to represent the material property variation seen between the implantation site and graft material and have evidenced little validation. Hence, there is a lack of understanding regarding which parameters are most important for the short-term mechanical stability of osteochondral grafts.

The aim of the study was to develop a method of integrating models of graft behaviour into a finite element (FE) model of a full tibiofemoral joint and provide an insight into the level of specimen specificity required for the model to be valid. There were three main objectives of the work, which each combined experimental and computational methods. The first was to develop an approach to accurately represent the inhomogeneous, specimen-specific bone properties of both the osteochondral graft and the surrounding host bone. The second was to understand and optimise the representation of the frictional forces between the graft and the host at the early stage of implantation, prior to osseointegration. The final objective was to implement these methods into a full tibiofemoral joint model to enable the mechanical performance of the graft to be evaluated.

## Materials and Methods

**Fig. 1:**
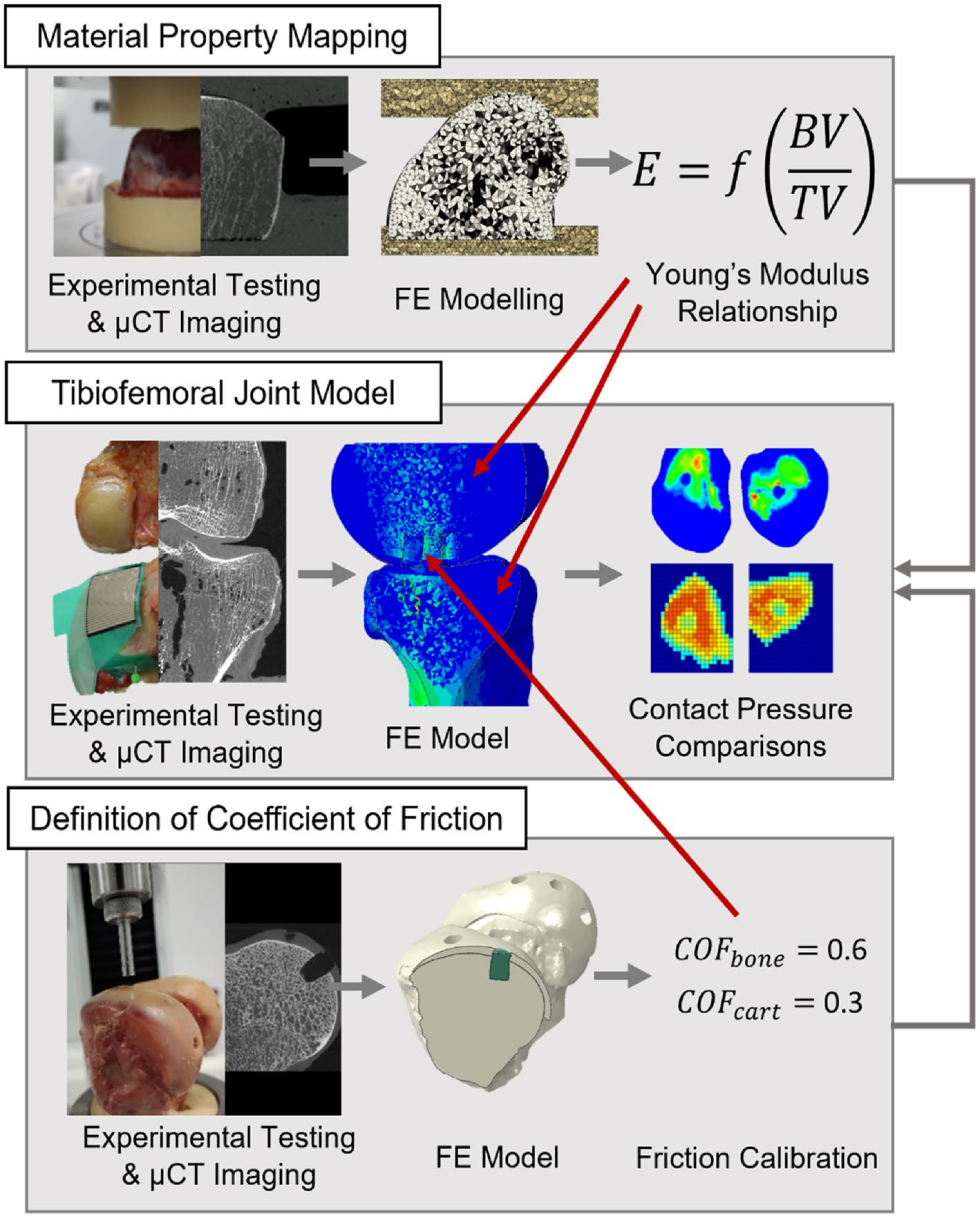
An overview of the three main arms of computational and experimental testing that were used in the study, including the origin of the coefficient of friction and material mapping properties that were used in the tibiofemoral joint model. Material property mapping and the definition of the coefficient of friction occurred in parallel, with values that were used in the tibiofemoral joint model

The methods to address each of the objectives are described below and an overview is given in Fig. 1. In all cases, mechanical testing was performed on a materials testing machine (Instron 3365 with a 5 kN load cell, Instron, UK) and specimens were imaged using micro computed tomography (µCT) on a HR-pQCT (XtremeCT, Scanco Medical AG, Switzerland; 82 µm isotropic voxel size: 900 lA, 60 kVp energy and 300 ms exposure time). Image segmentation and FE meshing were performed using Simpleware ScanIP (2021.03, Synopsis, USA) and FE models were all quasi-static nonlinear models solved using Abaqus (2017, Dassault Systèmes, France). Input files for the models are available in the associated dataset [20]***.*** The Concordance correlation coefficient (CCC) was used to quantify the degree of agreement or concordance between two arrays of variables, taking both precision and accuracy into account. The calculation to determine the CCC is described by Lin, [21].

Following ethical approval (East Midlands—Leicester South Research Ethics Committee (18/EM/0224)), a fresh-frozen human cadaveric knee was obtained and stored at − 40 °C for this study.

Further details on all computational methods can be found in the additional methods document in the associated dataset [20].

### Material Property Mapping Derivation

In order to implement inhomogeneous, specimen-specific material properties for the bone in finite element models, a material property mapping method developed in previous studies was used [22–24]. The mapping allows for a conversion between the bone volume fraction derived from 3D image data and the Young’s modulus of each finite element.

Experimentally, three human cadaveric knees were used. The femoral components were separated into individual condyles, the cartilage was removed, and they were potted in polymethylmethacrylate (PMMA; WHW Plastics, UK) cement endcaps. The six potted specimens were tested using a materials testing machine, with a load applied between two flat platens. A preconditioning [25], cyclic load to 100 N was applied 10 times, followed by loading to 1000 N or yield. A loading rate of 1 mm/min was applied to match the loading regime of the subsequent push-in and tibiofemoral joint loading tests. The stiffness of the samples was measured by taking the average gradient of the load-displacement curve between 400 and 600 N. This range was chosen because it gave comparable strains in the bone tissue to those in the FE models of the push-in tests and tibiofemoral joint model. A µCT scan of each specimen potted in its endcaps was taken prior to experimental loading.

**Fig. 2:**
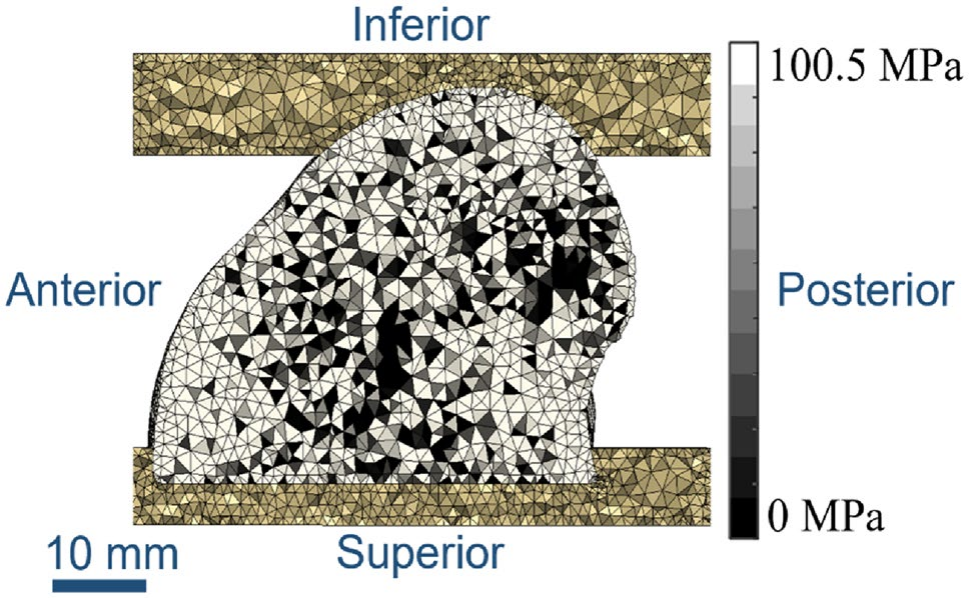
A mid-slice through the finite element mesh, showing PMMA endcaps at the top and bottom with the single condyle between. Element shading indicates bone density, with lighter regions indicating denser and therefore elements with a higher Young’s modulus

Finite element models were created from the µCT scan data based on a methodology developed and optimised previously for porcine tissue [24], Fig. 2. Briefly, the μCT background was first binarised, using a fixed threshold across all specimens, and was then downsampled to 0.164 mm^3^, such that the resulting voxel greyscale was proportional to the bone volume fraction (BV/TV). This made the image data more manageable to manipulate computationally and followed previously used methodology [24]. The bone was modelled as an isotropic linear elastic material, where the Young’s modulus of each element was proportional to the greyscale value of each voxel using a linear conversion factor [22, 26, 27]. Meshing utilised a target element edge length of 1 mm [24]. The high element density was required for the stability of subsequent models involving the osteochondral grafts. Given the element size dependence of the material property mapping, element size for the bone was kept consistent throughout the study. Previous mesh sensitivity tests comparing quadratic and linear tetrahedral elements found less than 1% difference when comparing stiffness and push-in forces [24]; hence, linear tetrahedral elements were used throughout. The PMMA endcaps were assigned a Young’s modulus of 2.45 GPa and a Poisson’s ratio of 0.3 [22]. The applied boundary conditions mimicked the experimental setup, using an encastre condition on the lower endcap and a kinematic coupling applied to the superior endcap where a 1-mm uniaxial displacement was applied.

The relationship between the Young’s modulus of each element and the bone density of the underlying voxel was optimised to minimise the root mean square difference between the FE-predicted stiffness and corresponding experimentally derived stiffness values of the six specimens. This calibration process was performed using the opti4abq optimisation toolbox [28] and has been documented previously [22, 27].

### Derivation of the Coefficient of Friction Values

Two frictional values between the graft and host were calibrated for the osteochondral graft models representing the behaviour of the cartilage-on-bone and bone-on-bone interfaces. The cartilage-on-cartilage contact was assumed to be frictionless according to the literature [18, 19, 29–31]. The two friction coefficients were optimised to maximise the concordance correlation coefficient between experiment and computational model across a set of push-in tests. These tests provide information on the stability of a graft at the early, pre-osseointegration, stage immediately after graft implantation.

Two cadaveric human distal femurs were used for a total of 12 push-in tests. Each femur received six osteochondral grafts into the femoral condyles, with the grafts taken from the trochlear groove. A surgical toolkit with a 6.35 mm diameter drill bit and 6.5 mm internal diameter chisel (Acufex Mosaicplasty, Smith & Nephew, UK) was used throughout the graft acquisition and implantation procedures. Three holes in each femoral condyle were drilled normal to the cartilage surface to a depth of 10 mm. Osteochondral grafts were taken from the trochlear groove of each femur and were trimmed to 10 mm in length, producing grafts with a 0.15 mm diameter oversizing compared to the drilled hole.

MicroCT scans were taken for the two knees in the native state, after graft harvest/hole creation, and after the push-in tests. A combination of the scans allowed for a determination of the graft/implant site position and bone properties of the grafts/implant site locations.

A material testing machine was used in conjunction with a rig that allowed the femur to be positioned such that the drilled hole was aligned with the loading direction [24], ensuring each push-in test was conducted along the graft axis. The grafts were inserted into the holes until flush with the surrounding cartilage surface with the aid of a tamp from the Acufex Mosaicplasty kit. A 6 mm indentor, attached to the head of the testing machine, was lowered until it was in contact with the graft surface. A displacement of 1 mm was then applied and the force–displacement data were recorded.

Computational modelling was carried out to mimic the experimental testing. The three scans of each knee were imported into Simpleware ScanIP, where they were registered using an automated process, with initial landmark selection. Segmentation and meshing followed the same steps as in the above sections.

Care was taken for the definition of the graft and drilled hole to ensure consistent FE model solution convergence, using registered pre- and post-harvest µCT scan data to define high-quality meshes [24]. Meshing settings matched those used in the above section. A hyperelastic, neo-Hookean material property was used for the host and graft cartilage [26], bulk modulus *K* = 16.67 MPa and shear modulus *G* = 1.37 MPa.

Contacts between the cartilage surfaces and the bone element sets used surface–to–surface hard contacts.

The model setup in Abaqus utilised a number of steps prior to the push-in load step to ensure that the initial positioning of the graft matched the experimental setup and that the pre-stress and strain due to the oversized graft were represented. The output from the model was the sum of the nodal reaction forces at 1 mm of displacement, which equated to the push-in force at 1 mm from the experiment. Remaining boundary conditions mimicked the experimental setup. The tangential friction values were calibrated by comparing the FE outputs to the experimental data using the concordance correlation coefficient.

### Tibiofemoral Joint Model

**Fig. 3:**
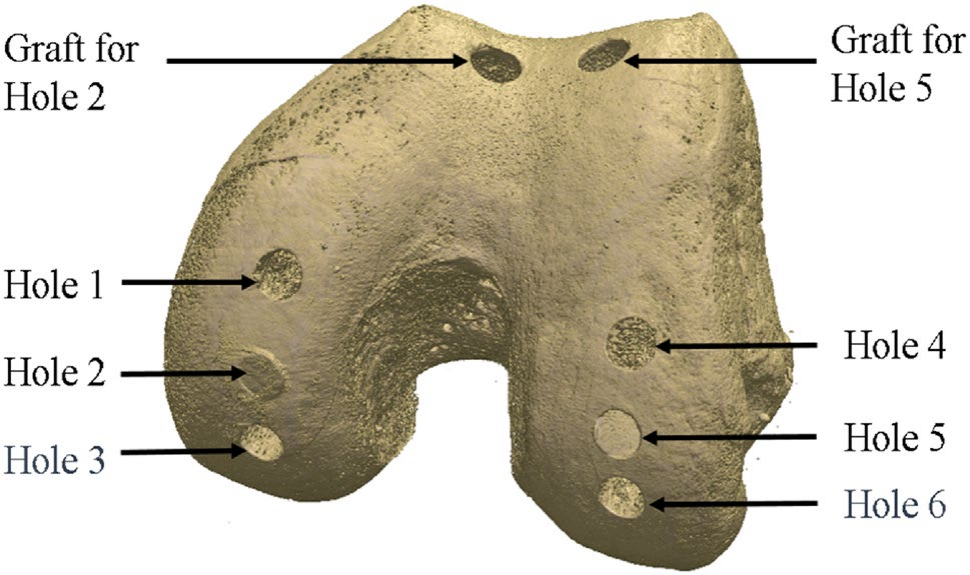
Rendering based on the microCT scan data showing the hole positioning and location of the graft harvest. Holes 1 and 4 remained as defects (drilled holes), Holes 2 and 5 received osteochondral grafts and Holes 3 and 6 received metal pins

A single cadaveric, human, tibiofemoral joint was used to develop a specimen-specific finite element model of that joint, containing an osteochondral graft repair. The sample had been tested previously in an intact state under quasi-static loading in a materials testing machine [32], and a corresponding FE model generated with comparisons made between the experimental and FE contact areas and shapes. In this study, six holes were drilled into the femoral condyles. Two holes remained unfilled, representing cartilage defects, two holes were repaired with osteochondral grafts taken from the trochlear groove, and two received stainless steel pins with matching geometry to the osteochondral grafts to represent a near-rigid implant, Fig. 3.

An experimental test rig, specifically designed for the investigation of contact mechanics in natural knee joints was used [33]. It was housed within a materials testing machine and allowed for the flexion angle to be changed by rotating the femur through the natural axis of rotation. The specimens were dissected by removing all soft tissues except for the cartilage. Both the femur and tibia were cemented in custom-built pots using PMMA following previously established techniques [34].

**Fig. 4:**
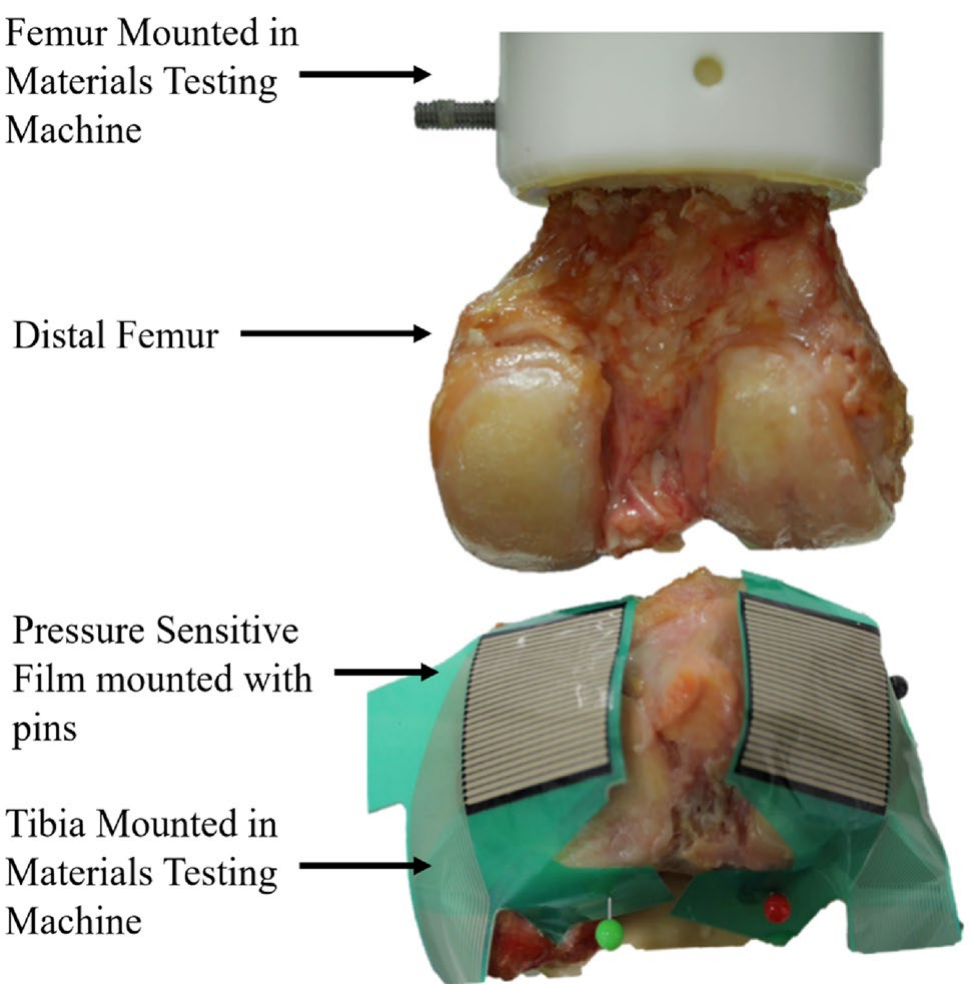
The experimental setup of the tibiofemoral joint model. The femur was potted and mounted to the head of the material testing machine and the tibia was potted and mounted to the base of the testing machine. Pressure sensitive film (Tekscan Pressure Mapping Sensor Model 4000) was fixed to the tibial plateau using pins and connected to a PC where the pressure was recorded

The defects were placed such that they could be loaded separately along their axes when the knee was aligned at 0, 10, and 30 degrees of flexion. The osteochondral grafts were inserted in the two 10-degree defects (Hole 2 and 5, Fig. 3) and metal pins at the two 30-degree defects (Hole 3 and 6, Fig. 3). Measurements were taken at these femoral angles in the intact state, after defect creation, and after graft/pin implantation, where relevant. An applied axial load was slowly ramped (1 mm/minute) up to 500 N and then held for 60 seconds, upon which the contact pressure data were recorded. Contact pressure measurements were obtained using pressure sensors (Tekscan Pressure Mapping Sensor Model 4000, Tekscan Inc., Boston, MA, USA), inserted between the cartilage layers and fixed using pins anteriorly and posteriorly, Fig. 4. Prior to testing, the pressure sensors were conditioned and then calibrated according to the manufacturer guidelines using the Tekscan software I-Scan against a pre-calibrated Instron 3365 load cell.

MicroCT scans of the knees were taken before testing, after graft harvest and defect creation, and after graft implantation (but before metal pin insertion, due to the effects of metal on CT scanning).

Finite element modelling of the knee used the same methodology to that used for the push-in test models. Mesh settings and target element edge length were also kept the same. Alignment of the knee used the PMMA pot around the femur as a straight edge to define the 10° and 30° flexion cases. The sides of the cement disc were aligned with one of the machine axes and could therefore be used to define femoral flexion. Material properties for the bone and cartilage from the previous section were used here, with the newly defined frictional properties applied. The articulating tibial and femoral cartilage surfaces were assigned frictionless surface-to-surface contact. An encastre boundary condition was applied to the inferior of the tibial bone and a kinematic couple applied between the top of the femoral bone and the centre of rotation. The computational graft implantation process matched that used in the previous section. Any changes to the flexion angle were then applied, relying of the interference fit between the graft and host to maintain the graft position. The 500 N load was then applied to the femur via the kinematic coupling. The contact pressure and contact area on the medial and lateral components of the tibial plateau were recorded for comparisons with the experimental cases.

## Results

The dataset associated with this study (3D images, experimental results, and computational models and scripts) is openly available from the University of Leeds data repository [20].

### Material Property Mapping

A conversion factor between the Young’s modulus of each element and the brightness of each downsampled voxel (which itself was proportional to the BV/TV) was derived for the six samples with a stiffness RMS error of 19%. The derived conversion factor was E = 100.5 × BV/TV (MPa). The mean experimental stiffness was 1514 N/mm (range from 1094 to 2608 N/mm).

### Push-in Testing and Coefficient of Friction Derivation

Experimentally, push-in forces at 1 mm of graft displacement ranged from 89 to 474 N with a mean of 219 N. The bone volume fraction of the grafts and host area varied considerably between the two knees and between host and graft site, Table 1. No correlation was found between the bone volume fraction of the graft and the recorded push-in force (*r*^2^ = 0.18). A weak correlation (*r*^2^ = 0.44) was found between the host site bone volume fraction and the push-in force.

**Table 1:** Bone volume fraction and experimental push-in force results for the two knees and test sites (mean value and standard deviation SD)

	Host site BV/TV (mean and SD)	Graft site BV/TV (mean and SD)	Push-in force (mean and SD)
Knee 1 (n = 6)	0.818 (0.044)	0.818 (0.025)	284 N (122 N)
Knee 2 (n = 6)	0.458 (0.028)	0.646 (0.050)	154 N (61 N)

**Fig. 5:**
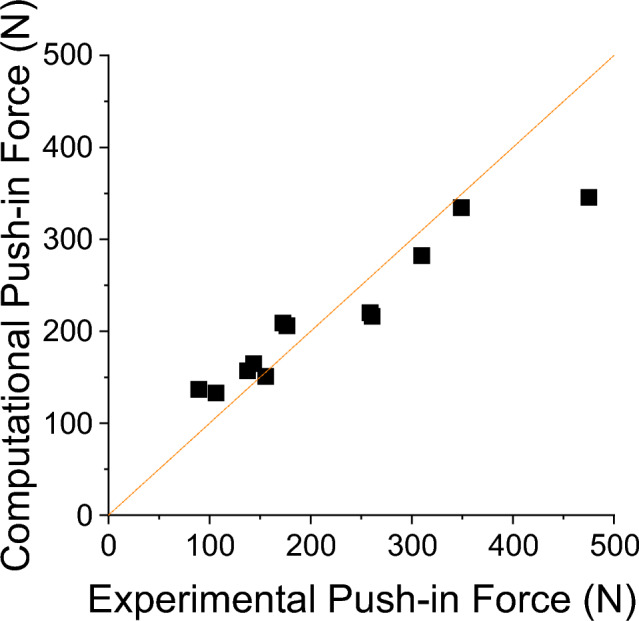
The 12 points correspond to the experimental and computational push-in force at 1 mm of graft displacement, measured experimentally using a materials testing machine and computationally using the sum of nodal reaction forces. Orange line indicates perfect agreement

Varying the coefficients of friction had little effect on the agreement between the experimental and computational results. Using the concordance correlation coefficient, the agreement ranged from worst agreement, 0.832 to best agreement 0.866. The optimal coefficients of friction for this model setup were a bone-on-bone coefficient of friction of 0.6 and a cartilage-on-bone coefficient of 0.3. The push-in test model agreement for the optimal properties is presented in Fig. 5. Tabulated results for all of the tested frictional values can be found in the associated dataset [20].

### Tibiofemoral Joint Model

**Fig. 6:**
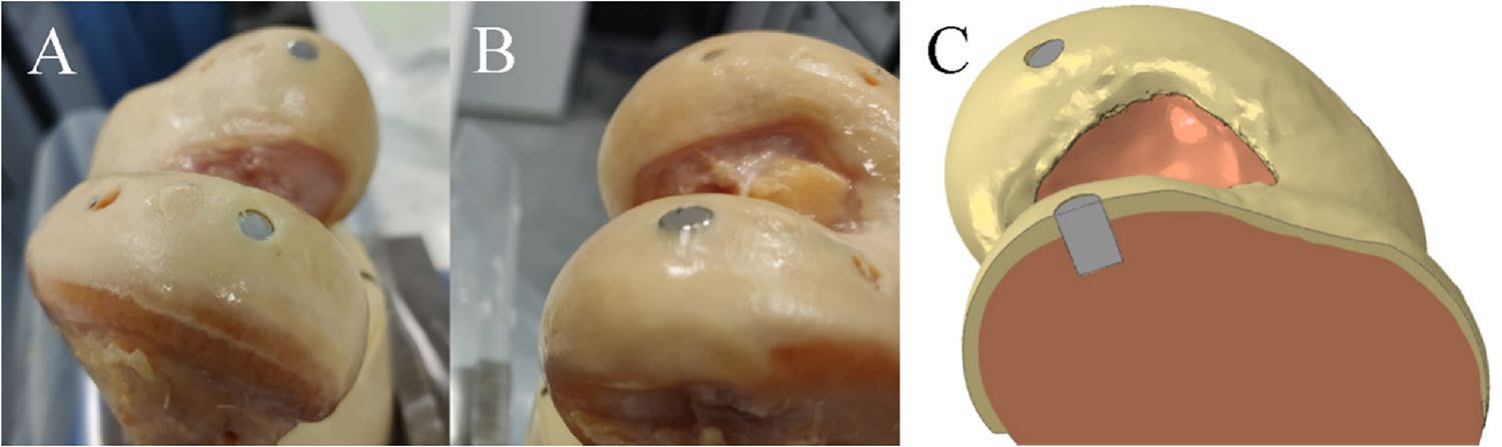
Experimental image showing the metal pin positioning between the below flush hole 3 pin (**A**) and the flush hole 6 pin (**B**).The matching finite element model (**C**) shows the two metal pins, the hole 3 pin (back) below flush and hole 6 pin (front) flush

Insertion flush with the surface was achieved in one osteochondral graft (hole 2) and one metal pin (hole 6, Fig. 6B), with the other graft and pin (Fig. 6A) sitting slightly below the surface.

**Fig. 7:**
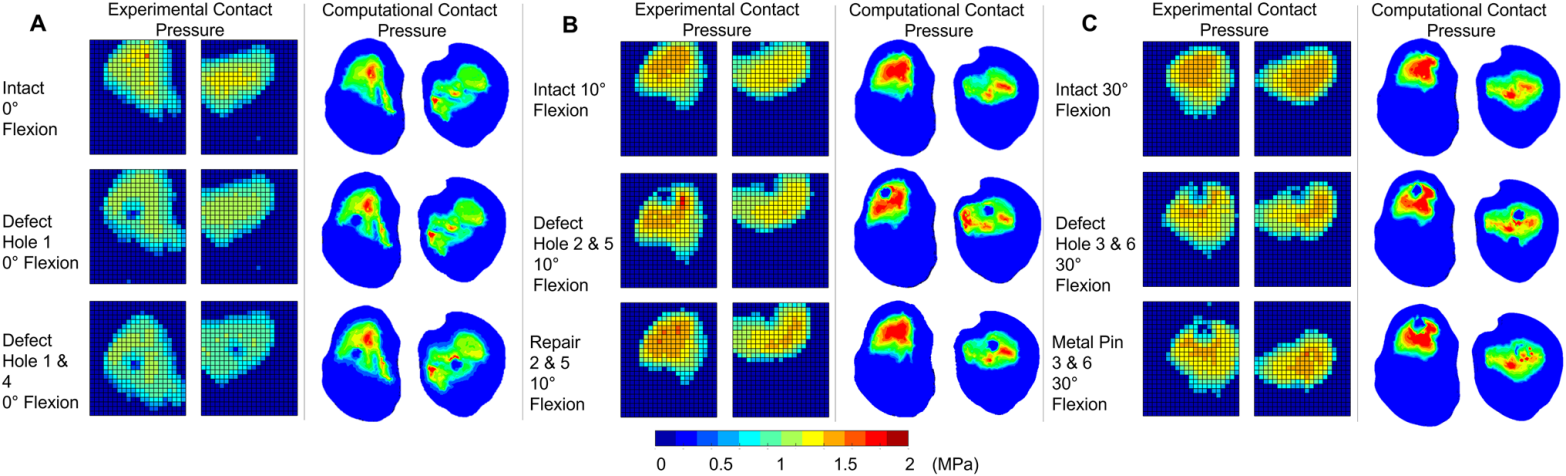
Experimental and computational contact pressure maps for the tibiofemoral joint model. Contact at 0°—**A**, 10°—**B** and 30°—**C**, flexion for three cases: intact, with lateral and medial condylar defect and with an osteochondral graft repair in both condyles

The tibial contact patch shape and load distribution matched well, from a qualitative perspective, between experiment and computational model for all flexion angles and different cases (Fig. 7). Intact cases, without defect or repair, were well matched between the computational and experimental contact pressure maps in terms of contact patch shape. Defect cases were clearly visible on the experimental pressure sensor maps and corresponded to a reduced or zero pressure value for the region. Hole size, measured from the experimental pressure maps, was approximately 5 sensels in diameter, in terms of reduced or zero contact pressure sensels. This corresponded to a diameter of 6.35 mm (1.27 mm sensel pitch) and equalled the size of the drill bit used. Computational defect size was modelled explicitly as 6.35 mm. The position of the defect with respect to the contact patch matched closely between the computational and experimental cases along both the x and y axes. Repair and metal pin cases were accurately represented in the computational pressure maps, for when the graft/pin was inserted flush (Hole 2 & 6) and when the graft was inserted below flush (Hole 3 & 5). The flush metal pin was indistinguishable from the intact case or an osteochondral graft repair in both the experimental and computational pressure maps.

**Fig. 8:**
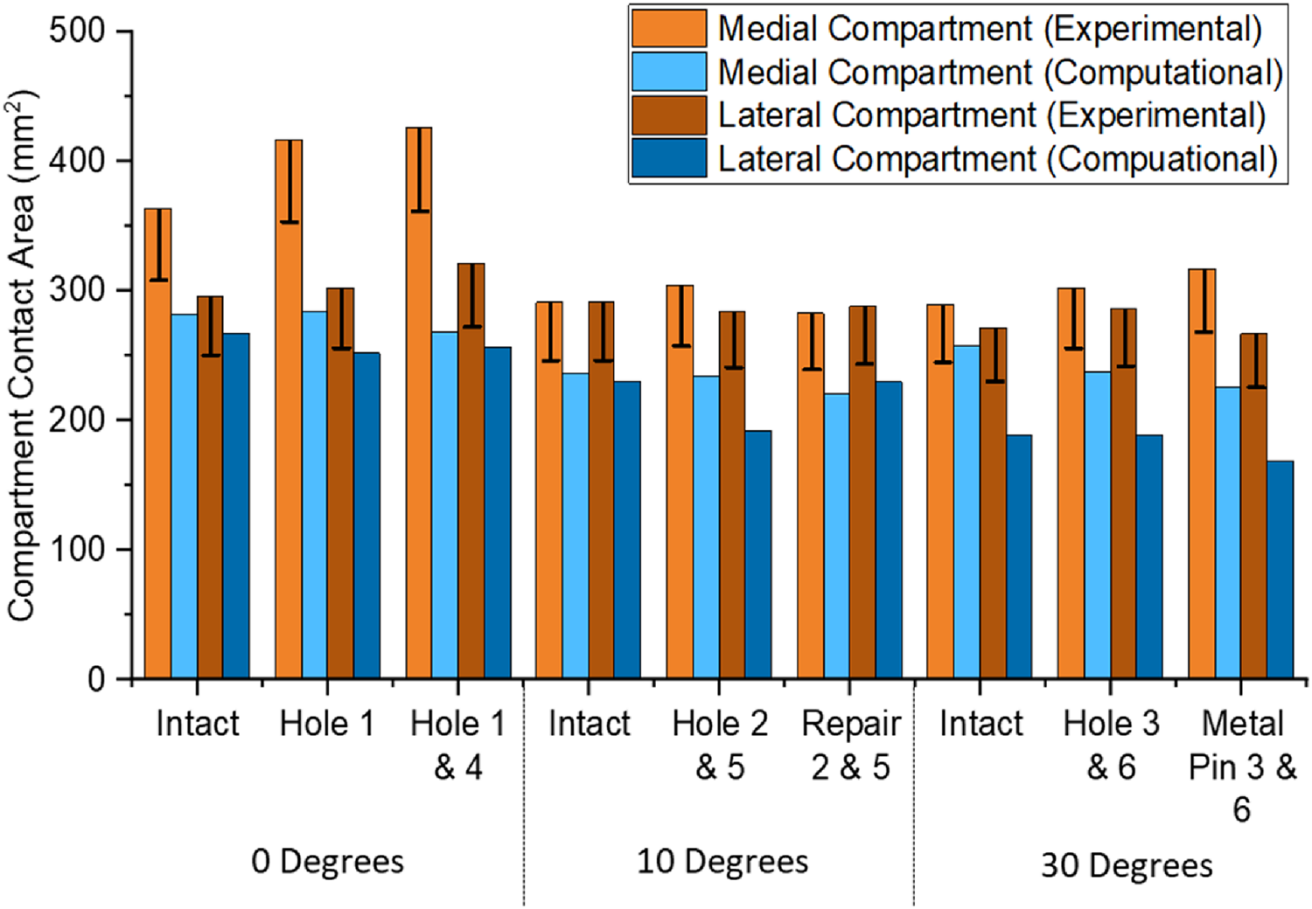
Lateral and medical compartment contact area results for the nine test cases, comparing between experimental and computaional results. Error bars represent one of the sources of error—the inability to count partial contact across pressure sensor sensels

Contact area comparisons, Fig. 8, showed a consistent underestimation of approximately 25% in the computational models; however, the dominant compartment, in terms of contact area, remained consistent between experimental and computational results. Experimental error for contact area was related to the number of sensels at the contact patch edge and ranged from 45 to 54 sensels for the smallest to the largest contact patch. This equated to 72.6 mm^2^ to 87.1 mm^2^ of potentially overestimated area equivalent to 13% to 17% of the reported value.

## Discussion

In this study, a method of modelling osteochondral grafts within a statically loaded tibiofemoral joint was developed. The method relies on a detailed description of the geometry and positioning of the osteochondral grafts, independently calibrated material properties for the ranges of strain that are expected within the model, and a calibrated set of frictional values between the graft and the host materials. These properties have been captured and calibrated in this study to provide a model that is suitable for a parametric analysis of osteochondral grafting techniques.

One purpose of these models is to assess the effect of mismatch between the bone density of the grafts and that of the surrounding host bone. Evidence from porcine bone studies demonstrated varying relationships between bone density and push-in force [24]. Specifically, the correlation (*r*^2^ = 0.48) between these variables in the graft from porcine bone starkly contrasts with a notably lower correlation (*r*^2^ = 0.18) found in the present study. Inversely, when examining host bone relationships, the porcine bone demonstrated a relatively low-density–force relationship (*r*^2^ = 0.19) in comparison with the more robust relationship observed in this study (*r*^2^ = 0.44). The latter, a somewhat stronger correlation between the host bone density and push-in force, implies that bone quality at the graft site's base might bear more significance than the graft material properties themselves. This discrepancy is likely attributable to divergences between porcine and human bone types: the former features a near-uniform distribution of trabecular bone and includes a growth plate, while the latter experiences a notable decline in density across the 1 to 2 cm region beneath the cortical shell. The push-in force ranges of similar studies [14, 35, 36] all fall within the ranges measured here, when forces at 1 mm of displacement below flush are compared.

A mapping constant between the bone volume fraction in an element and the Young’s modulus was found to be comparable to those used in other studies for similar methodologies [22] and for other studies modelling similar human trabecular bone [19, 37, 38]. The RMS error was notably high; nonetheless, this is consistent with the variability in human tissue properties, namely bone density, trabecular structure, and mineral density, as corroborated by findings from other researchers [39] and comparative analyses between human and alternative mammalian bone samples [40].

Computational models of the push-in tests showed excellent agreement to the experimental results in terms of force data. This provides confidence in the model that the mechanical surface interaction of the graft is being accurately represented, that any displacements of the grafts under joint loading will be reliable and that the effects of different scenarios will be identifiable. There was a general insensitivity to the coefficient of friction values between the contact surfaces with the maximum difference in the mean push-in force across all coefficients of friction values tested being 25 N. Importantly, this only produced a difference in the CCC of 0.034, an effect much smaller than other sources of variation such as the material properties of the bone or cartilage. Trabecular bone coefficient of friction values has large ranges, as reported in the literature [41]; however, the value calibrated in this study fits well within the experimentally derived range [41].

Assessment of contact pressure distributions enabled assessment of how effectively a graft has restored the intact articular cartilage surface contact mechanics, highlighting areas of increased or decreased pressure which may be detrimental to the cartilage tissue. The depiction of the hole was well represented, with the dimensions of the hole accurately portrayed in the contact pressure maps. Both computational and experimental methods were sensitive enough to detect when the graft was slightly below flush compared to the surrounding cartilage and could show restoration when the graft was flush. It is worth noting that due to the pressure sensor material, the depiction of the hole was not always recorded as a clear absence of pressure, instead a reduction in the pressure compared to the intact case. Validation of the computational prediction is complicated by the limitations of the experimental equipment. It is possible that the apparent underestimation of contact area by the computational model was actually an overestimation by the experimental setup. For example, in the experiment, the contact areas were derived through a summation of the non-zero grid elements of the pressure sensor, and therefore, the full area of a sensel is counted even where there is only partial contact across its region. The error shown in Fig. 8 is therefore a minimum error due to partial sensel contact and does not account for other, non-circumferential partial contact, nor other sources of experimental error.

The osteochondral graft repair at hole 2 restored the contact pressure map back to that of the intact case and was well matched in the FE model. While a restoration in contact pressure can be classified as a success, a risk of subsidence remains through: repeated loading, loading at other flexion and adduction/abduction angles, and the application of larger loads. The models are suited to investigate a range of these cases, including loading at static points across a gait cycle and repeated loading to investigate subsidence, with a caveat that current models would not include damage or plasticity-based subsidence and increased loading points would greatly increase computational cost.

Drill depth (11.1 mm) and graft length (10 mm) mismatch was found at hole 5, which led to the graft being inserted below flush of the surrounding cartilage and resulted in a minor change to the medial cartilage contact pressure in both experiment and FE model. For the FE model, the graft had undergone subsidence of approximately 0.5 mm despite starting initially below the flush level. This shows clear sensitivity to graft length and hole depth and is compounded with the relationship between the BV/TV of the host bone and the push-in force. This sensitivity shows that the models are well suited for investigating the effects of bone quality at the bottom of the hole and graft/hole length mismatch.

Using a metal pin instead of the osteochondral graft resulted in no measurable difference when compared to the osteochondral graft and, when implanted flush with the surrounding cartilage surface, restored the pressure distribution to that of the intact case. This suggests that the contact mechanical assessment methods used here are insensitive to the material properties, at least for this particular case, and that a more detailed examination of the graft-host interaction is required. Such investigations into properties for the graft would ensure that the graft maintains its position until the bony components are fully integrated and is well suited to a FE model investigation.

In summary, a method has been developed to model osteochondral grafts in human femoral condyles and has been shown to be successful in representing both osteochondral push-in tests and tibiofemoral joint loading behaviour. The method relies heavily on the inclusion of inhomogeneous, specimen-specific bone properties to represent push-in forces or early graft stability. Conversely, little sensitivity was found for the coefficients of friction between the different contacting materials. The model is considered suitable for the assessment of bone density mismatch between the graft and the host bone, and the effects of articular surface restoration. The approach can now be used to examine a range of clinical scenarios with different auto- or allograft configurations.
